# Commentary: Azathioprine as an adjuvant therapy in severe Graves’ disease: a randomized controlled open-label clinical trial

**DOI:** 10.3389/fendo.2024.1342915

**Published:** 2024-02-21

**Authors:** Madhukar Mittal, Azher Rizvi

**Affiliations:** Department of Endocrinology and Metabolism, All India Institute of Medical Sciences Jodhpur, Jodhpur, Rajasthan, India

**Keywords:** Graves’ disease (GD), azathioprine (AZA), anti thyroid drugs, remission, TRAb thyroid antibodies

## 1 Introduction

We read the study by Allam and colleagues with interest, which studied the role of azathioprine as an adjuvant therapy to anti thyroid drugs (ATD) in patients with moderate and severe Graves’ disease (GD) in a randomized controlled trial. The primary outcome of rate of remission after a follow up period of 2 years was compared among three randomized groups of patients, one on conventional therapy with ATDs and the other 2 on different doses of azathioprine in addition to conventional therapy (1). Currently recommended therapies for GD do not address the underlying immunopathogenesis of the disease and addition of immunosuppressives to standard treatment has been shown to improve relapse rates (2). Several immunotherapies including biologics, small molecule peptides and immunomodulators have shown efficacy in preliminary studies and could potentially transform the management of GD (3).

## 2 Inconsistencies in remission criteria

The results of the study are striking, with a high remission rate of 87.5% among patients taking adjuvant azathioprine therapy (1). The American Thyroid Association (ATA) uses a remission criteria of being euthyroid for at least 12 months after withdrawing ATDs (4). The authors here use a criteria for remission which includes complete (standard remission criteria) and partial remission (defined as being euthyroid while being on 5-15 mg/day of carbimazole for at least 12 months even with positive TSH receptor antibodies). There seems to be no explanation given for using an add-on partial remission criteria and this combined remission definition is inconsistent with previous literature including the guidelines by ATA and other thyroid associations (4, 5).

## 3 Unusually low remission rate among patients receiving conventional therapy with ATD

Considering the long term remission after a course of ATDs has been reported to be around 50%, with a range of 30-70% (6), it is certainly curious to note the extremely low remission rate of 7.4% observed among the group receiving ATDs alone, after 2 years of follow up. Interestingly enough, about 31% of patients with GD have been reported to have spontaneous remission even without ATDs (7). This remarkable divergence of the remission rate from not only previous studies but also our own clinical experience makes us wonder as to the basis of such low remission rates seen with ATD use in this study. The authors do report an additional 25.9% of patients which the authors consider to be in partial remission but with continued use of ATDs.

## 4 Conflicting findings with different doses of azathioprine used

This was the first study to evaluate the effect of specific doses of azathioprine on outcomes as previous studies with azathioprine in patients with GD and Graves’ orbitopathy (GO) have not evaluated it (8, 9). The authors however do not address the possible reasons for the discordant results seen with the different doses of azathioprine used. Complete remission was reported in 87.8% of patients in the group which received azathioprine at doses of 1mg/kg/day as adjuvant therapy to ATD with none of the patients reported to have partial remission. In contrast, complete remission rate in the group which received a higher dose of adjuvant azathioprine (2mg/kg/day) was 68.9% with an additional 24.4% of patients reported to have achieved partial remission. No explanation is provided for these observed differences.

## 5 No clarity regarding the duration of follow up in the study

In the article published, at several points in the text the authors mention that the follow up period was 12 months. Yet the data provided in table 2 as well as in the Kaplan Meier survival curve is for a follow up period of 2 years after remission. These varying follow up periods mentioned at different places need more clarification so as to remove any contradictions and bring out more clarity for the readers.

## 6 Discussion and conclusion

We appreciate the efforts of Allam and colleagues in studying a commonly used, well tolerated and economical immunosuppressive agent to complement the management of GD. Future comparisons of the frequency of exacerbations and new onset of Graves’ ophthalmopathy would allow a better comparison of the immunosuppressive effects of additional azathioprine administration.

In this study, subjects were randomly assigned using a computer program, matched for age, sex and TRAb. However, thyroid volume varied considerably among the three groups, although the differences were not significant. It would be useful to perform a multivariate analysis, such as proportional hazards analysis or nominal logistic analysis, to eliminate the effects of confounding to determine the factors associated with recurrence. The statistical methods used in this study should also be mentioned.

The concerns emphasized in our commentary lead us to critique certain aspects of the study methods and seek certain plausible answers or explanations for the points raised above.

## Author contributions

MM: Conceptualization, Writing – review & editing. AR: Writing – original draft.
